# Oridonin Ameliorates Concanavalin A-Elicited Hepatitis in Mice: Insight into Suppressing TLR7/PKM2/NLRP3-Driven Inflammation and M1/M2 Polarization

**DOI:** 10.3390/jox16040138

**Published:** 2026-07-28

**Authors:** Saif Dhahir, Fatma M. Amin, Manar A. Nader

**Affiliations:** 1 Department of Pharmacology and Toxicology, Faculty of Pharmacy, Mansoura University, Mansoura 35516, Egypt; saifdh19@gmail.com (S.D.); manarahna@mans.edu.eg (M.A.N.); 2 Department of Pharmacology and Toxicology, Faculty of Pharmacy, Mansoura National University, Gamasa 7731168, Egypt

**Keywords:** ORI, AIH, Con A model, M1/M2 polarization, TLR7, PKM2, NLRP3

## Abstract

**Background:** Autoimmune hepatitis (AIH) represents a clinically challenging immune-mediated liver disease, owing to its complex pathogenesis and limited targeted therapeutic options. Growing evidence highlights the key role of lymphocyte-mediated hepatic inflammation, dysregulated cytokine milieu, and oxidative stress in AIH progression. Oridonin (ORI), a bioactive diterpenoid with well-established antioxidant, anti-inflammatory, and immunoregulatory effects, remains unexplored in AIH. The present work aims to survey the potential impacts of ORI in Concanavalin A (Con A)-prompted AIH in mice, with particular emphasis on TLR7/PKM2/NLRP3 inflammatory signaling and macrophage polarization. **Methods:** Male BALB/c mice (*n* = 30) were allocated into five groups: CTR group, ORI-CTR group, Con A group, ORI (5 mg/kg) + Con A group, and ORI (10 mg/kg) + Con A group. ORI was administered i.p. for 4 days before a single Con A injection (15 mg/kg i.v.). Serum liver enzymes, hepatic pathological changes, oxidative milieu, and varied immunological factors were assessed. **Results:** ORI markedly attenuated Con A-induced hepatic injury, as evidenced by the reduced liver transaminases, preserved hepatic architecture, and restored oxidative milieu. Moreover, ORI suppressed T-cell activation, modulated M1/M2 polarization, and reduced pro-inflammatory cytokines. These effects were accompanied by the suppression of TLR7/PKM2/NLRP3 inflammatory signaling. **Conclusions:** ORI exhibits hepatoprotective effects against Con A-induced AIH and these effects are associated with the modulation of inflammatory and immunometabolic responses as well as downregulating TLR7/PKM2/NLRP3 signaling.

## 1. Introduction

Autoimmune hepatitis (AIH) is a chronic inflammatory condition of the liver that may progress to cirrhosis and liver failure if not properly managed [1,2]. The global annual incidence and prevalence of AIH are estimated at 1–2 and 20 cases per 100,000 people, respectively [3]. Interestingly, infliximab has been used as a rescue treatment and the effectiveness of this drug has recently been confirmed in a multicenter case series [4].

The pathogenesis of AIH is multifactorial, implicating the infiltration of T cells and macrophages into the periportal liver regions, which triggers inflammatory responses that damage hepatocytes. Dysregulated immunoregulatory mechanisms such as CD8+ cytotoxicity, auto-antibodies production, and imbalanced M1/M2 macrophage polarization are key contributors to AIH development and progression [3,5]. Moreover, the nucleotide-binding domain and leucine-rich-containing pyrin 3 (NLRP3) inflammasome has been involved in the pathogenesis of AIH, where its activation promotes the release of pro-inflammatory cytokines, aggravating liver inflammation and hepatocyte injury [6]. Recently, the M2 isoform of pyruvate kinase (PKM2) has been identified as a chief nonmetabolic regulator of NLRP3 that moderates pyroptosis stimulation in macrophages and offers insights for future therapeutic strategies for acute liver failure (ALF) [7].

Continuing studies on the pathogenesis of AIH have led to the development of innovative therapeutic approaches. Despite the advances in the treatment of AIH [3,8], AIH presents complex diagnostic and therapeutic challenges.

Oridonin (ORI) is a tetracyclic diterpenoid isolated from the Chinese traditional medicinal herb *Rabdosia rubescens*. It has been consumed as a principal component in traditional Chinese medicine for thousands of years because of its various pharmacological potential uses [9]. Recently, ORI was found to exert numerous valuable biological effects, such as anti-inflammatory [10,11], anti-cancer [12], and immunoregulation effects [13].

The anti-inflammatory potential of ORI through macrophage regulation has been reported [11]. In addition, ORI elicited protection against carrageenan-induced pleurisy through attenuating the release of cytokines and neutrophil infiltration, as well as oxidative milieu [14]. Interestingly, ORI ameliorated liver ischemia/reperfusion (IR) injury by suppressing the NLRP3-mediated macrophage proptosis [15]. Recent evidence has further highlighted the hepatoprotective role of ORI through the modulation of hepatic macrophage responses. A recent study demonstrated that ORI attenuated experimental liver injury by regulating hepatic macrophage activation and suppressing ROS/NLRP3 signaling, thereby reducing inflammatory responses and tissue damage. These findings further support the ability of ORI to target macrophage-associated inflammatory pathways and reinforce its potential role in liver diseases characterized by excessive immune activation and inflammasome signaling [16]. In addition, ORI secured against LPS/D-galactosamine-provoked acute liver injury in mice by reducing liver damage via the inhibition of TNF-α production [17] and the inhibition of the NLRP3 inflammasome [18]. These outcomes reveal that ORI may serve as a probable therapeutic agent for a diversity of inflammation-linked diseases, with NLRP3 as the direct target for mediating its anti-inflammatory activity [10]. Furthermore, the literature suggests that ORI prompts macrophage polarization through different cellular signaling pathways; however, the precise molecular mechanisms governing this regulation remain incompletely understood [19].

The Concanavalin A (Con A)-prompted liver injury model is widely used as an experimental representation of AIH [20]. The hepatotoxic effects of Con A are primarily mediated through the recruitment of T cells and macrophages, which triggers the upregulation of pro-inflammatory cytokines, leading to ALF [21]. The Con A experimental model, as a T-cell determined model, better mimics the mechanism and features of clinical AIH, enabling research on the pathogenesis of AIH [22,23,24].

Considering the aforementioned, this work was envisioned to survey the impending mechanisms triggering the possible hepatoprotective effects of ORI on Con A-provoked AIH in mice in relation to the suppression of TLR7/PKM2/NLRP3 signaling and modulating M1/M2 polarization.

## 2. Materials and Methods

### 2.1. Drugs and Chemicals

ORI and Con A were purchased from Sigma-Aldrich, St. Louis, MO, USA. Both Con A and ORI were prepared in normal saline (0.9% *w*/*v*) immediately prior to administration to the mice. All other chemicals were of analytical purity.

### 2.2. Animals and Ethical Approval

Thirty male BALB/c mice (5–7 weeks old; 30 ± 5 g) were used in this study. Animals were maintained under specific pathogen-free conditions at 24 ± 2 °C with 55 ± 8% relative humidity and a 12 h light/dark cycle, with free access to food and water. The animals were allowed a 2-week acclimatization period. All experimental procedures were approved by the Animal Care and Use Committee of Mansoura University (MU-ACUC) (Protocol No. PHARM.MS.24.10.120).

### 2.3. Experimental Protocol

Animals were allocated to experimental groups using a computer-produced randomization sequence. To minimize potential bias, investigators responsible for histopathological examination and immunohistochemical assessment were blinded to group allocation until completion of data collection and analysis (*n* = 6/each group):

**Control (CTR) group:** Normal mice received normal saline (i.p.) for four consecutive days and i.v on the fourth day.

**ORI-CTR group**: These mice were given ORI (10 mg/kg, i.p) for four consecutive days [18].

**Con A group**: These mice were challenged with Con A (15 mg/kg; i.v. in the tail vein) on the fourth day of the experiment [25].

**ORI 5 + Con A group**: These mice were treated with ORI (5 mg/kg, i.p.) [18] for 4 consecutive days; then, on the fourth day of the experiment, Con A was injected 2 h after administration of ORI.

**ORI 10 + Con A group**: These mice were given ORI (10 mg/kg, i.p.) [18] for 4 consecutive days. Con A was injected 2 h after administration of ORI on the fourth day of the experiment.

Conspicuously, ORI was administered via i.p. injection over a period of four days to ensure consistent systemic administration during the acute phase of Con A-induced hepatitis. The administered dose and route of administration (i.p.) were selected based on previous preclinical studies [18,26]. Although the i.p. route does not fully simulate clinical routes, i.p. administration is widely used in experimental studies, mainly with drugs with limited oral bioavailability, such as ORI. ORI has low oral bioavailability and poor water solubility and undergoes first-pass metabolism [27]. Future studies will explore clinically relevant administration routes and optimized delivery systems.

Additionally, regarding the 4 day pre-treatment regimen, it was selected based on a previous study in our lab [28], for studying the potential protective and immunomodulatory effects against the early immune-mediated hepatic injury induced by Con A.

### 2.4. Specimen Collection

Following Con A injection (3 h later), mice were anesthetized using secobarbital (50 mg/kg, i.p.). This time point (3 h) was selected based on previous reports demonstrating that Con A-induced hepatitis is characterized by rapid activation of T lymphocytes and macrophages, accompanied by early cytokine production and initiation of hepatocellular injury within the first few hours after administration. Therefore, the 3 h interval was considered suitable for assessing the early inflammatory and immune-mediated events targeted in the present study. The blood was drawn from the retro-orbital plexus and centrifuged at 2000 rpm for 15 min. The serum obtained after centrifugation was immediately employed for hepatic function markers. The liver was dissected and washed with cold 0.9% normal saline after being brought out of deep anesthesia. A portion of the first lobe of liver tissue was fixed with neutral-buffered formalin and used for histopathological examination and further immunohistochemical (IHC) staining. The other section in the specimen was washed using 2.5% glutaraldehyde to assess the transmission electron microscope (TEM). The remaining lobes of the liver were employed to prepare 10% *w*/*v* liver homogenate in cold phosphate-buffered saline (PBS; 0.01 M, pH 7.4). The homogenate was next subjected to centrifugation (4000 rpm, 15 min, 4 °C) using Sigma3K30 HS (Sigma Laborzentrifugen GmbH, Osterode am Harz, Germany), and the supernatants were used for determination of biochemical markers.

### 2.5. Determination of Liver Enzymes

Serum levels of AST and GGT were spectrophotometrically measured using the diagnostic kits (Agappe Diagnostics LTD., Kerala, India).

### 2.6. Histopathological Examination

The liver lobes were preserved in 10% buffered formalin. Thereafter, they were washed with 70% alcohol, stirred and mixed in a magnetic stirrer, and again dried at different concentrations of alcohol (range from 70 to 99%), then embedded in paraffin. The paraffin blocks were cut into sections (5 μm thickness) with a rotary-type microtome. Sections were mounted onto glass, laid out on slides, and allowed to dry overnight. After H&E staining, histopathological changes in the slides were observed under a light microscope (Olympus CH2, Olympus Corporation, Tokyo, Japan). Data were reviewed by a blinded pathologist to examine different histopathological changes such as ballooning, fatty change, apoptotic bodies, inflammatory infiltrate location, extent and nature of the invading cells, and vascular changes (sinusoidal dilatation in addition to congestion). Multiparametric semiquantitative histopathological scoring was assessed in six randomly selected fields per slide, using a scale ranging from 0 to 3 according to the severity of lesions. The evaluation included assessment of hepatic injury alterations, the extent of inflammatory reactions, hepatocellular necrosis, and degeneration. Each parameter was scored individually, and the total histopathological score for each group was obtained by summing the individual parameter scores [29].

### 2.7. TEM Examination

Small pieces of liver tissue (about 1 mm^3^) were immediately fixed in 2.5% glutaraldehyde diluted with 0.1 M phosphate buffer (pH 7.4) at 4 °C overnight. After the primary fixation, specimens were rinsed with phosphate buffer and then post-fixed in 1% osmium tetroxide for 2 h. After that, the tissues were dehydrated in a series of graded ethanol (30–100%) and infiltrated with epoxy resin. Ultrathin sections (70 nm in thickness) were prepared with an ultramicrotome and mounted on copper grids. They were stained with uranyl acetate and lead citrate for contrast enhancement, and electron micrographs were taken with a transmission electron microscope operating at an accelerating voltage of 80 kV. Quantitative assessment of ultrastructural alteration, including mean degenerated mitochondria, mean number of lipid droplets per hepatocyte, mitochondria, lipid, and nucleus diameter, was done using ImageJ Software (Fiji ImageJ 1.54p, National Institutes of Health (NIH), Bethesda, MD, USA) [30].

### 2.8. Oxidative Stress Markers

Oxidative stress factors in the hepatic tissue were also determined. Lipid peroxidation was determined as malondialdehyde (MDA) using an established color developed with thiobarbituric acid reactive substances at 532 nm [31]. Hepatic antioxidant power was determined: GSH level, according to the previously described method [32], and total antioxidant capacity (TAC), using colorimetric kits (Biodiagnostic, Giza, Egypt and MyBiosource, San Diego, CA, USA, respectively).

### 2.9. Biological Assay of Different Markers Using Enzyme-Linked Immunosorbent Assay (ELISA)

Cluster of differentiation (CD)-4, (CD)-8, Immunoglobulin G (IgG), Tumor necrosis factor (TNF)-α, (CD)-163, Interferon (INF)-γ, (CD)-80, (CD)-86, Nucleotide-binding domain, leucine-rich–containing family, pyrin domain–containing-3 (NLRP3), Toll-Like Receptor 7 (TLR7), and Interleukin 1 Beta (IL-1β) were measured utilizing the corresponding kits and were assessed according to corresponding manufacturer instructions as shown in Supplementary Table S1.

### 2.10. Western Blotting Analysis

After extraction of total protein using ReadyPrep protein extraction kits (Bio-Rad Laboratories Inc., Hercules, CA, USA), the protein concentration in each tissue sample was determined using the Bradford protein assay kit; 20 µg protein per sample was separated by gel electrophoresis on SDS-polyacrylamide gel and then transferred to PVDF membrane. Membrane blocking was performed in Tris-buffered saline with Tween 20 (TBST) and 3% bovine serum albumin (BSA) at room temperature for 1 h. The blotted aim for proteins incubated overnight at 4 °C with specific primary antibodies against PKM2 (Thermo Fisher Scientific/Invitrogen, (Waltham, MA, USA), Cat# PA5-28700), TLR7 (Thermo Fisher Scientific/Invitrogen, (Waltham, MA, USA), Cat# ER30606), NLRP3 (Thermo Fisher Scientific/Invitrogen, (Waltham, MA, USA), Cat# PA5-79740), and CD206 (MR6F3) (Thermo Fisher Scientific/Invitrogen, (Waltham, MA, USA), eBioscience Catalog # 12-2061-82). Then, membranes were probed with horseradish peroxidase (HRP)-conjugated secondary antibody (Novus Biologicals, Centennial, CO, USA). The chemiluminescent substrate (Clarity ^TM^ Western ECL substrate (Bio-Rad Laboratories, Hercules, CA, USA)) was applied to the blot according to the manufacturer’s recommendation. The chemiluminescent signals were captured using a CCD camera-based ChemiDoc™ MP imaging system (Bio-Rad Laboratories, Hercules, CA, USA). Image analysis software was used to read the band intensity of the target proteins against the control sample β-actin (housekeeping protein) by protein normalization on the ChemiDoc™ MP imager (Bio-Rad Laboratories, Hercules, CA, USA).

### 2.11. Immunohistochemistry (IHC) Analysis

Hepatic protein expressions of CD80, CD86, and iNOS were evaluated via IHC staining according to Karadimas and colleagues. Liver slices were deparaffinized and hydrated, and H_2_O_2_ was applied to inhibit endogenous peroxidase activity. Antigen retrieval and 5% bovine serum albumin (BSA) blocking was conducted; biotin-conjugated rabbit anti-CD80, CD86, and iNOS (Abcam, Cambridge, UK, ab64116; Servicebio, Hubei, China, GB113109 & Servicebio, Hubei, China, GB11119, respectively) were used overnight (4 °C). The antiserum was detected with biotin-conjugated goat anti-rabbit IgG and streptavidin-peroxidase complex and then visualized by 3,3′-diaminobenzidine (DAB). Sections were examined using an Olympus microscope. A monochrome image representing the DAB content was then subjected to frequency analysis using ImageJ software (FIJI, National Institutes of Health, USA), and the calculation of the area fraction of DAB (antigen) was done. IHC examinations were recorded in 4 sections per slide. Three photos were taken per section.

### 2.12. Quantitative Real-Time PCR of CD86 and CD206 mRNAs

Total RNAs were isolated from liver tissues using TRIzol reagent (Invitrogen, Waltham, MA, USA). One μg of total RNA was reverse-transcribed to cDNA using QuantiTects reverse-transcription kit (Qiagen, Germantown, MD, USA). The gene expressions of target genes in the cDNAs obtained were quantified using Maxima SYBR Green/Fluorescein qPCR Master Mix (Thermo Scientific, Rockford, IL, USA), primer pairs (Supplementary Table S2), and a Rotor Gene Q thermocycler (Qiagen, Hilden, Germany). The relative expression of each target gene with respect to the internal control, GAPDH mRNA, in the same sample was calculated using the ΔΔCt method [33].

### 2.13. Statistical Analysis

Data were analyzed using the Statistical Package for the Social Sciences (SPSS version 25.0; IBM/SPSS Inc., Chicago, IL, USA). Results were presented as mean ± standard deviation (SD). Normality and homogeneity of variances were assessed using the Shapiro–Wilk and Levene’s tests, respectively. For comparisons among groups, one-way ANOVA was performed, followed by Tukey’s honestly significant difference (Tukey-HSD) post hoc test to identify pairwise group differences. Non-parametric data were analyzed using the Kruskal–Wallis test, followed by Dunn’s multiple-comparison post hoc test. A *p*-value < 0.05 was considered statistically significant.

## 3. Results

As no significant differences were observed between the ORI-CTR and control groups regarding the serum biochemical parameters and histopathological findings, the ORI-CTR group was not included in the subsequent molecular analyses. This approach was adopted to focus on the primary objective of evaluating the protective effects of ORI against Con A-induced autoimmune hepatitis while avoiding unnecessary data redundancy.

### 3.1. Impact of ORI (5 and 10 mg/kg) on Liver Enzyme Activity

As presented in Table 1, Con A treatment triggered a marked hepatocellular injury as evidenced by the significantly increased AST (*p* < 0.001) and GGT (*p* < 0.001) activities, compared to CTR. The pre-treatment with ORI (5 and 10 mg/kg) significantly attenuated (*p* < 0.001) the Con A-induced elevation in serum enzyme activity in a dose-dependent manner.

### 3.2. Impact of ORI (5 and 10 mg/kg) on Histopathological Analysis

**Figure 1:**
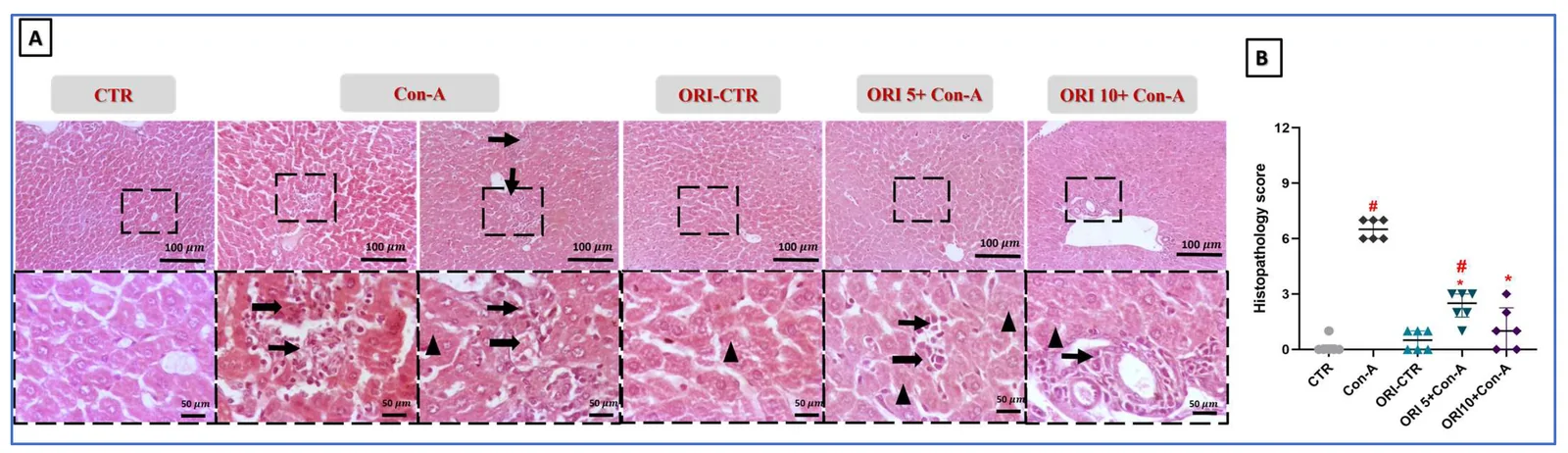
(**A**) **H&E-stained liver micrographs showing Con A-induced hepatocellular damage and modulation by ORI (5 and 10 mg/kg)**. (**B**) **The corresponding quantitative assessment of hepatic histopathological scores among different treatment groups.** Values were presented as median± interquartile range, with the Kruskal–Wallis test followed by Dunn’s multiple-comparison post hoc test, *n* = 6. # * Significant compared to CTR and Con A, respectively. CTR, Control; Con A, Concanavalin A; ORI, Oridonin; thin arrows, inflammation; thick arrows, hepatocellular necrosis; and arrowheads, hepatocellular vacuolation. Image magnification: 100× = Bar 100 μm, 400× = Bar 50 μm.

Regarding the liver architecture in response to Con A and ORI (Figure 1), staining with H&E revealed that liver sections in both CTR groups showed the normal histological appearance of hepatic parenchyma. The ORI-CTR group showed approximately normal hepatic parenchyma except for occasional tiny intracytoplasmic vacuoles. In contrast, stained-liver sections from the Con A group showed scattered hepatocellular necrosis characterized by a shrunken, hyperesinophilic cytoplasm with a pyknotic or karyorrhectic nucleus with mild degeneration characterized by swollen hepatocytes with vacuolation beside multifocal inflammatory aggregations of moderate numbers of lymphocytes, plasma cells, macrophages, and few neutrophils (Figure 1). The liver sections from ORI 5 + Con A-treated mice revealed few to mild instances of hepatocellular vacuolation with multifocal portal mild to moderate inflammatory aggregations. For the sections from ORI 10 + Con A-treated mice, few instances of hepatocellular vacuolation and occasional hepatocellular necrosis surrounded by few inflammatory cells were revealed.

### 3.3. Impact of ORI (5 and 10 mg/kg) on TEM Analysis

**Figure 2:**
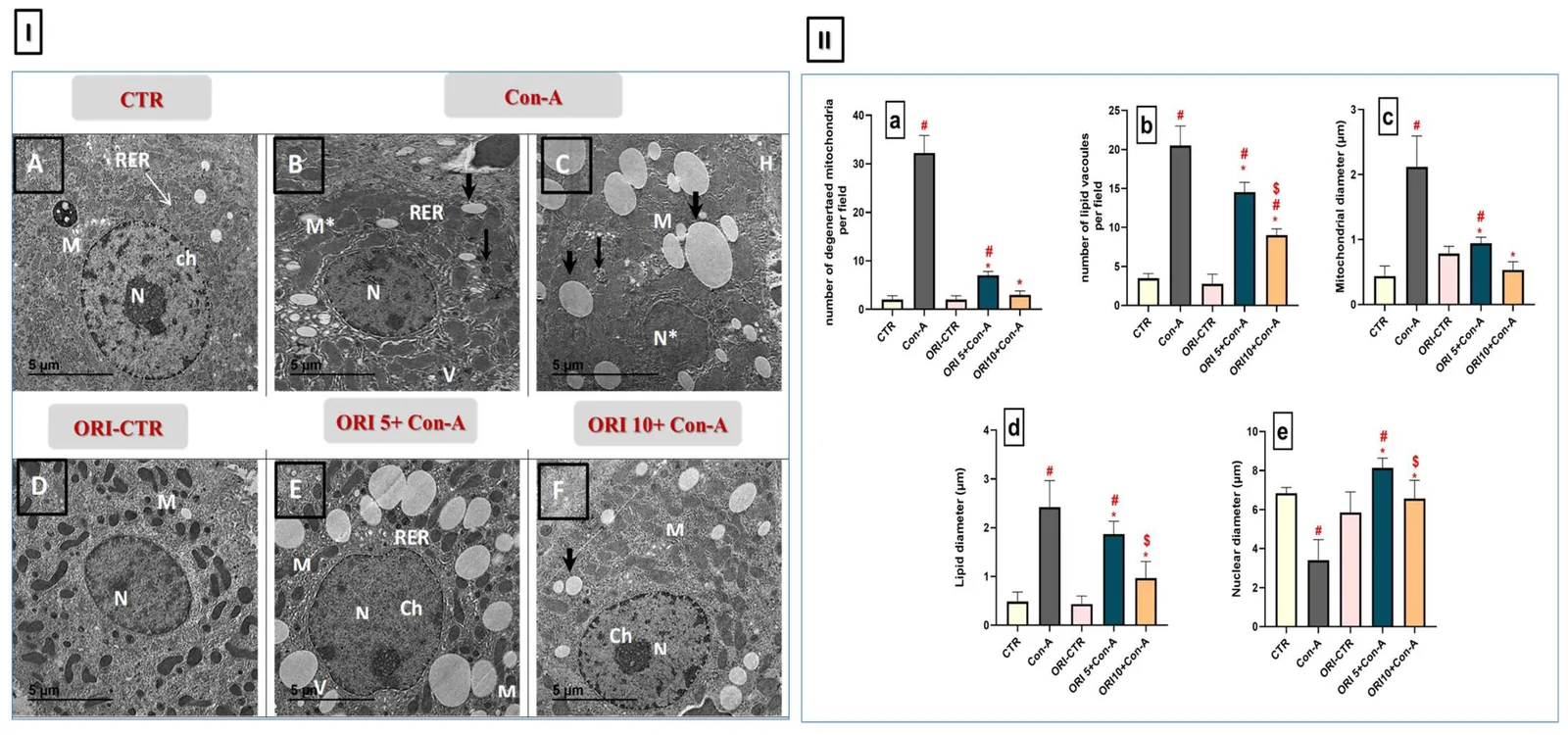
**(I) Transmission electron micrographs of the liver sections from studied groups**: (**A**) CTR, (**B**,**C**) Con A, (**D**) ORI-CTR, (**E**) ORI 5 + Con A, and (**F**) ORI 10 + Con A. (**II**) **The corresponding quantitative analysis of ultrastructural changes in hepatocytes**: (**a**) number of degenerated mitochondria, (**b**) number of lipid vacuoles per hepatocytes, (**c**) mitochondrial diameter, (**d**) lipid, and (**e**) nuclear diameter. Values were presented as mean ± SD. Data were obtained by one-way ANOVA followed by Tukey–Kramer multiple comparisons, *n* = 5. # * $ Significant compared to CTR, Con A, and ORI 5+ Con A, respectively. CTR, Control; Con A, Concanavalin A; ORI, Oridonin; CH, Heterochromatin; RER, rough endoplasmic reticulum; N, normal nuclei; N*, altered nuclei; M, normal mitochondria; M*, swollen electron dense mitochondria; thick arrow, presence of multi-lipid droplets; thin arrow, numerous lysosomes; and white arrow, normal shaped mitochondria. 80× and one follicular cell layer scale bar.

TEM micrographs of the liver in the experimental groups were shown in Figure 2. The hepatocellular architectural profile of the CTR and ORI-CTR groups was normal, having large oval nuclei, a normal nuclear border, no loss of cellular boundaries, numerous mitochondria, and well-arranged endoplasmic reticulum (ER). On the other hand, various degenerative changes in hepatocytes in the Con A group were detected as shrunken and pyknotic nuclei, mitochondrial pleomorphism with a loss of cristae and swelling, a dilated rough ER containing involved lysosomes, and multiple lipid droplets distributed throughout their cytoplasm that were observed in apoptotic and necrotic stimuli. Moreover, pre-treatment with ORI preserved hepatocyte morphology. The nuclear and cytoplasmic structures were close to those of healthy cells, with minor swelling of mitochondria, and few existing majorities (lipid droplets in elasticity) by a low dose (5 mg/kg). A high dose (10 mg/kg) effectively restored the normal hepatocyte ultrastructure, which had well-preserved mitochondria with occasional degenerated ones and rough endoplasmic reticulum profiles with less fat accumulation.

### 3.4. Impact of ORI (5 and 10 mg/kg) on Hepatic CD4, CD8, and IgG

**Figure 3:**
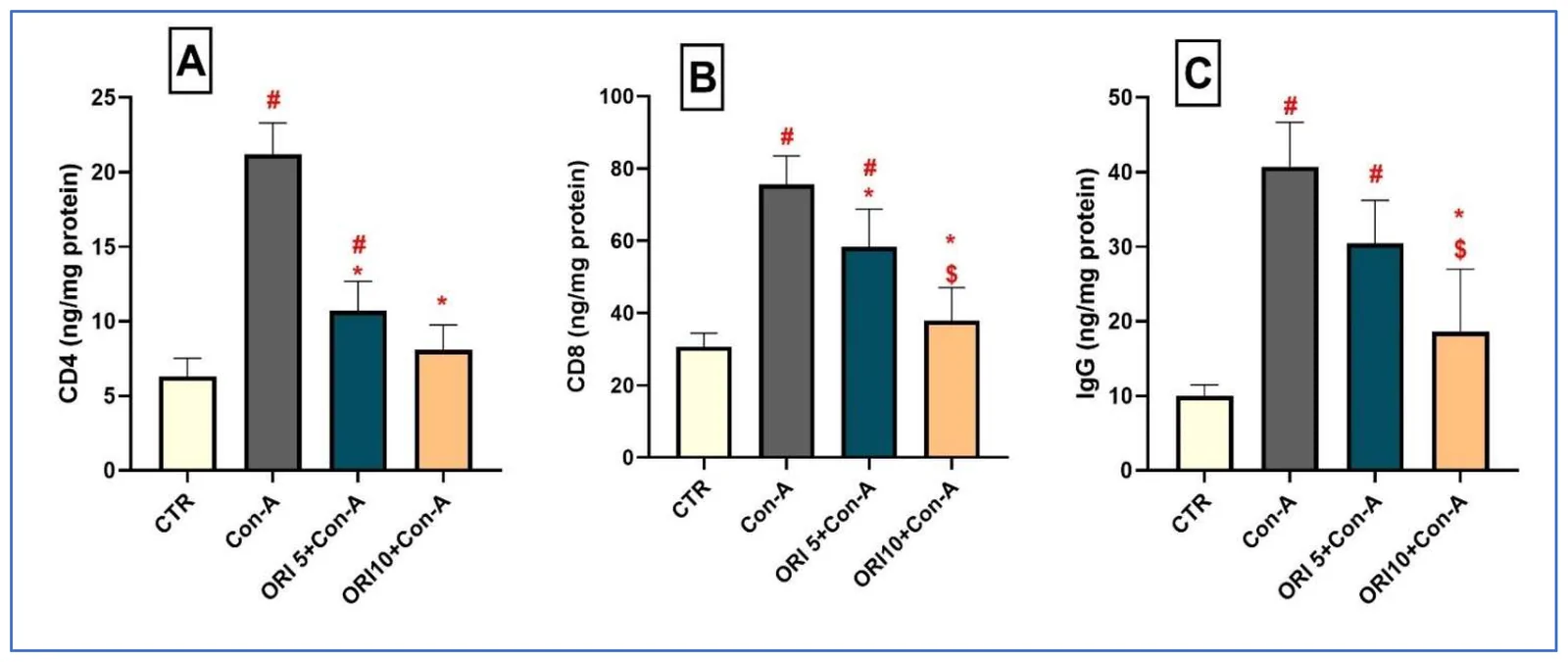
**Impact of ORI (5 and 10 mg/kg) on Con A-induced changes in hepatic CD4** (**A**)**, CD8** (**B**), **and IgG** (**C**) **protein contents.** Values were presented as mean ± SD. Data were obtained by one-way ANOVA followed by a Tukey–Kramer multiple comparison test, *n* = 5. # * $ *p* < 0.05 compared with CTR, Con A, and ORI 5 + Con A, respectively. CTR, Control; Con A, Concanavalin-A; ORI, oridonin; CD4, Cluster of differentiation 4; CD8, Cluster of differentiation 8; and IgG, Immunoglobulin G.

CD4 and CD8 were selected as markers of T-cell activation and infiltration because T lymphocytes are key mediators of autoimmune liver injury and Con A-induced hepatitis. The hepatic CD4, CD8, and IgG levels in the experimental groups were shown in Figure 3. T-cell-related parameters showed a significant increase (*p* < 0.0001) in the Con A-treated group versus CTR values, as well as IgG values, indicating potential immune activation upon hepatic injury caused by Con A. ORI pre-treatment dose-dependently modulated immune responses (*p* < 0.0001), with low (5 mg/kg) and high (10 mg/kg) doses restoring the Con A-induced elevations in CD4, CD8, and IgG, suggesting its immunoregulatory role in hepatoprotection.

### 3.5. Impact of ORI (5 and 10 mg/kg) on Hepatic CD80 and CD86

**Figure 4:**
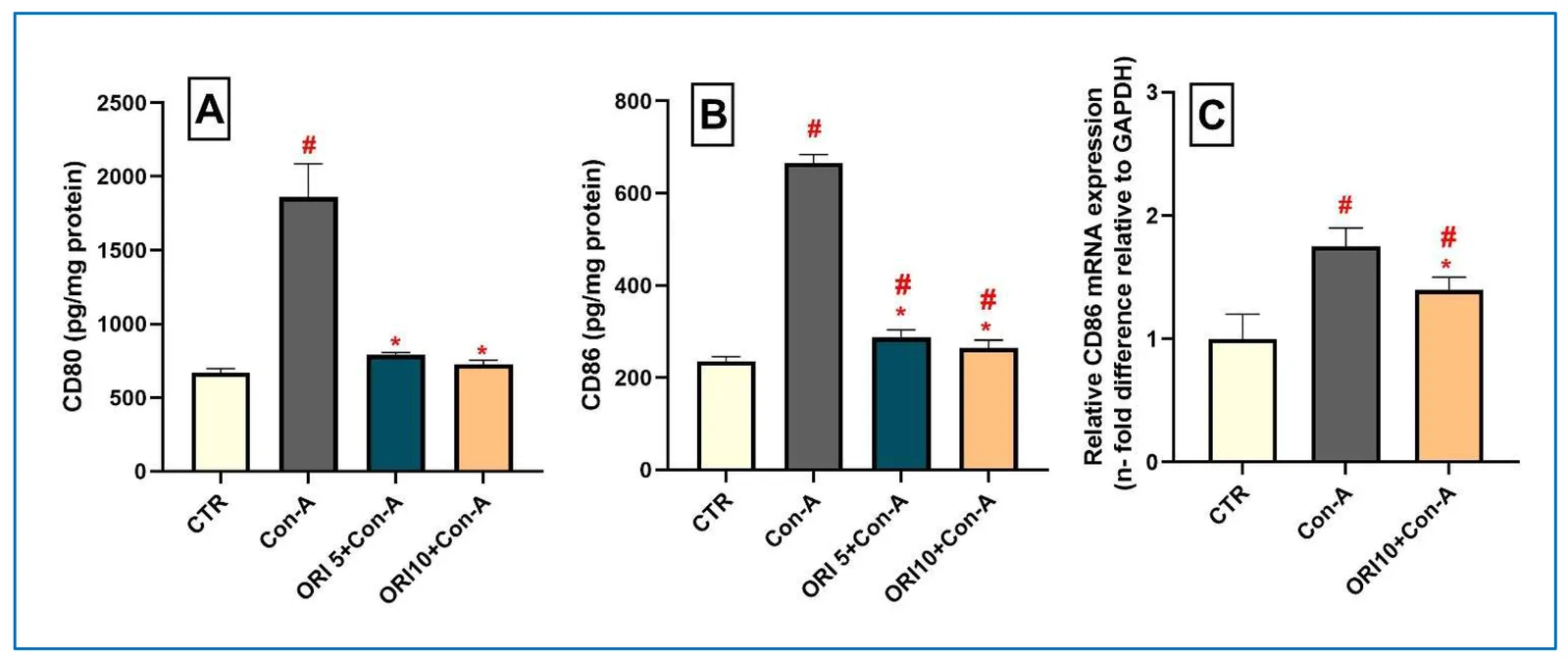
**Impact of ORI (5 and 10 mg/kg) on Con A-induced alterations in hepatic M1-related markers: CD80** (**A**) **and CD86** (**B**) **protein level, and** (**C**) **mRNA expression.** Data were presented as mean ± SD from one-way ANOVA followed by Tukey–Kramer method for multiple comparison, *n* = 3–5. # * *p* < 0.05 compared with CTR and Con A, respectively. CTR, Control; Con A, Concanavalin A; ORI, oridonin; and CD80 and CD86, Cluster of Differentiation 80 and 86.

CD80 and CD86 were evaluated as markers associated with pro-inflammatory macrophage activation and co-stimulatory signaling, which play important roles in antigen presentation, T-cell activation, and the amplification of hepatic inflammatory responses. As demonstrated in Figure 4, the hepatic protein levels of CD80 and CD86 and mRNA expression of CD86 (Figure 4C) were remarkably increased (*p* < 0.0001) in the Con A group compared to the CTR group. ORI dose-dependently reduced these elevations in protein levels (*p* < 0.0001). Additionally, ORI at a high dose (10 mg/kg) attenuated the escalated mRNA expression of CD86. These results indicate that ORI mediates macrophage polarization through the suppression of M1-related pro-inflammatory markers.

**Figure 5:**
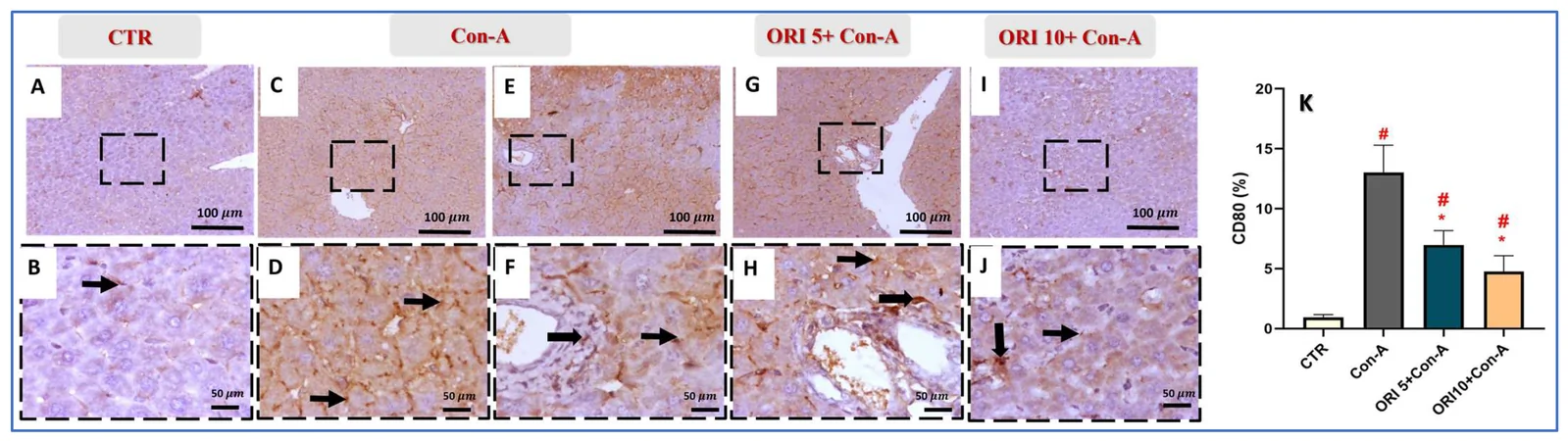
**Immunohistochemical analysis of the impact of ORI (5 and 10 mg/kg) on CD80 protein expression (IHC) in the Con A model.** (**A**,**B**) CTR group showing absent CD80 expression in hepatocytes except faint expression in rare Kupffer cells. (**C**–**F**) Con A showing diffuse intense expression in activated Kupffer cells and periportal inflammatory cells. (**G**,**H**) ORI 5 + Con A showing mild to moderate immunopositive-stained periportal inflammatory cells with scattered positivity in Kupffer cells. (**I**,**J**) ORI 10 + Con A showing few immunopositive-stained Kupffer cells and inflammatory cells. Thin arrows, positive Kupffer cells; thick arrows, positive inflammatory cells. Image magnification: 100× = Bar 100 μm, 400× = Bar 50 μm. (**K**) Semi-quantifications of % area protein expression of CD80 in the studied groups. Data were represented as mean ± SD using one-way ANOVA followed by Tukey–Kramer multiple comparisons, *n* = 5. # * Significantly different compared to CTR and Con A, respectively. CTR, Control; Con A, Concanavalin A; ORI, oridonin; and CD80, Cluster of Differentiation 80.

**Figure 6:**
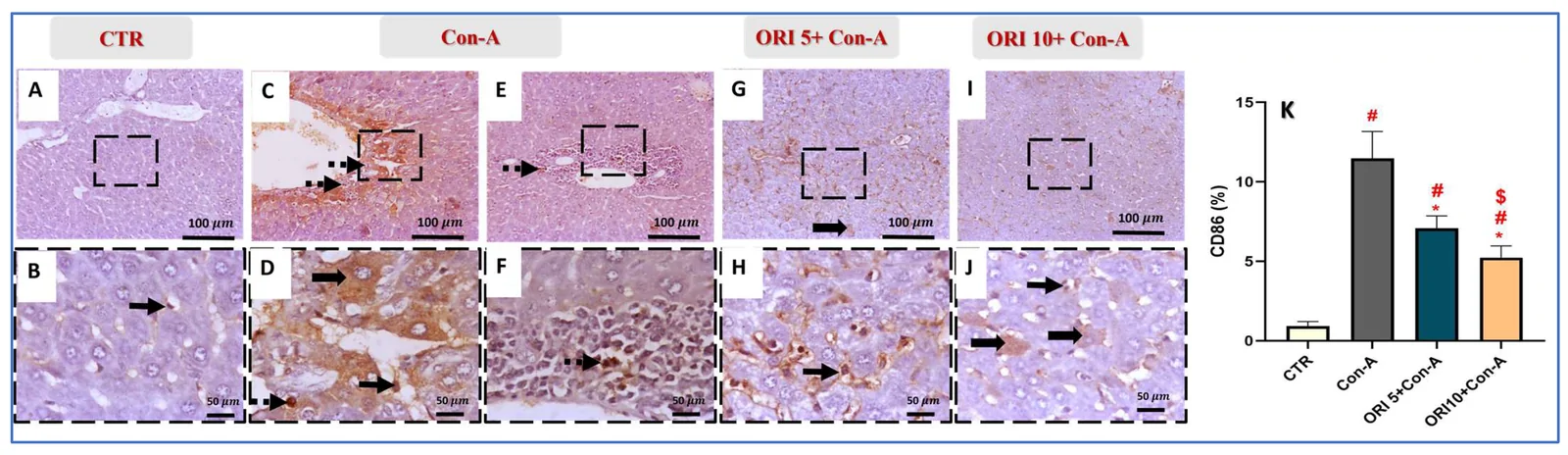
**Immunohistochemical analysis of the impact of ORI (5 and 10 mg/kg) on CD86 protein expression (IHC) in the Con A model.** (**A**,**B**) CTR group showing negative expression in hepatocytes with little expression in Kupffer cells. (**C**–**F**) Con A showing focal to coalescing perivascular intense cytoplasmic expression in hepatocytes and invading inflammatory cells. (**G**,**H**) ORI 5 + Con A showing moderate expression in activated Kupffer cells with little expression in hepatocytes. (**I**,**J**) ORI 10 + Con A showing few to mild immunopositive cytoplasmic-stained hepatocytes and Kupffer cells. Thin arrows, positive Kupffer cells; thick arrows, positive hepatocytes; dashed arrows, positive invading inflammatory cells, mostly macrophages. Image magnification: 100× = Bar 100 μm, 400× = Bar 50 μm. (**K**) Semi-quantifications of % area protein expression of CD86 in the studied groups. Data were represented as mean ± SD using one-way ANOVA followed by Tukey–Kramer multiple comparisons, *n* = 5. # * $ Significantly different compared to CTR, Con A, and ORI 5 + Con A, respectively. CTR, Control; Con A, Concanavalin A; ORI, oridonin; and CD86, Cluster of Differentiation 86.

Additionally, an immunohistochemistry analysis also verified the induction of M1-related markers CD80 (Figure 5) and CD86 (Figure 6) after Con A treatment. The level of expression (%) of both CD80 and CD86 were strongly enhanced (*p* < 0.0001) in Con A hepatic tissues, compared to CTR. Pre-treatment with ORI dose-dependently decreased (*p* < 0.0001) the expression of both markers (Figure 5K and Figure 6K, respectively).

### 3.6. Impact of ORI (5 and 10 mg/kg) on Hepatic Expression of CD163, CD206, IFN-γ, TNF-α, and iNOS

**Figure 7:**
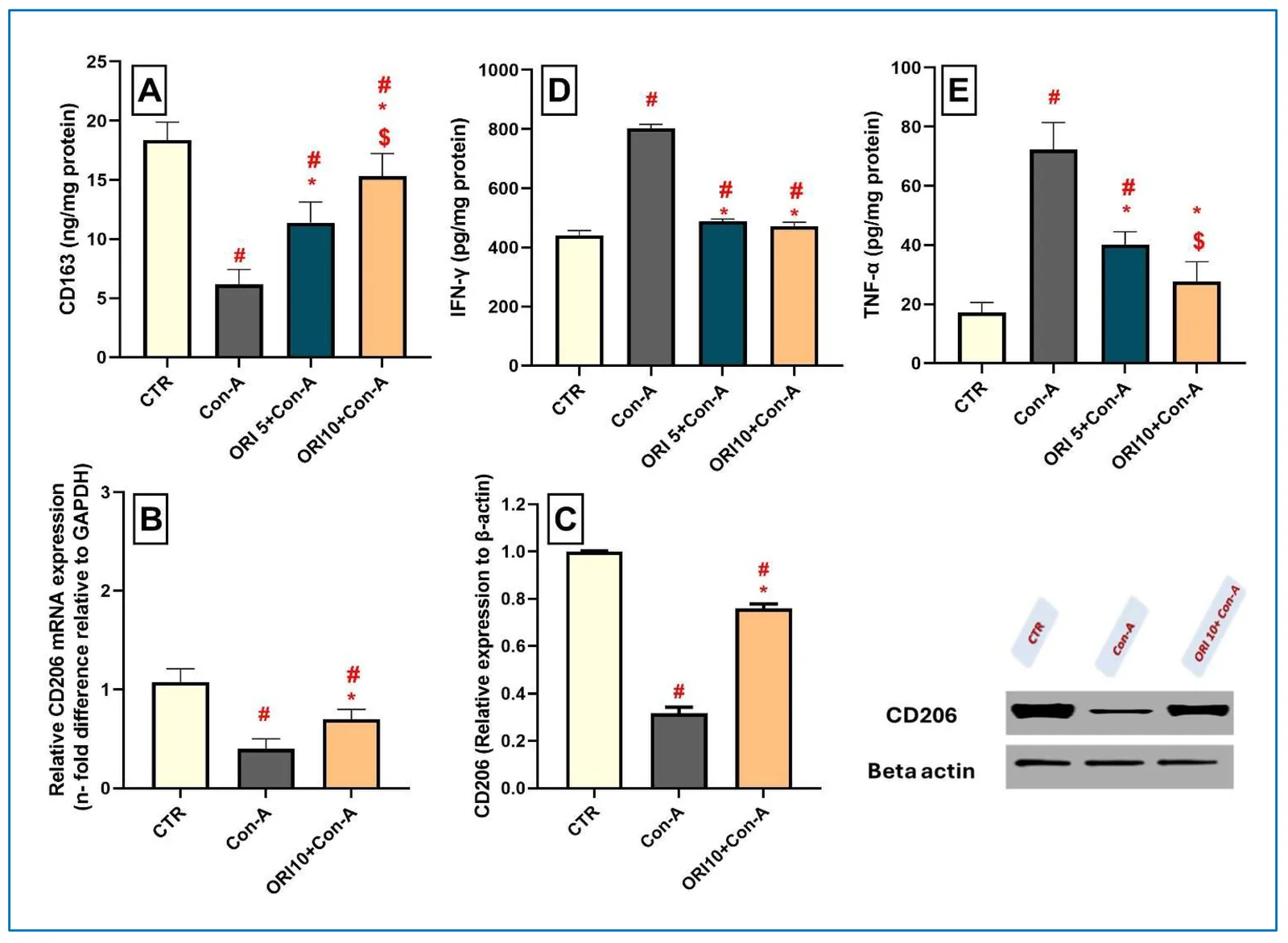
**Impact of ORI (5 and 10 mg/kg) on Con A-induced variations in the hepatic CD163** (**A**) **and CD206** (**B**) **mRNA expression, protein expression and Western blot bands** (**C**)**, IFN-γ** (**D**)**, and TNF-α** (**E**). Data were represented as mean ± SD using one-way ANOVA followed by Tukey–Kramer multiple comparisons, *n* = 3–5. # * $ Significant difference in relation to CTR, Con A, and ORI 5 + Con A, respectively. CTR, Control; Con A, Concanavalin A; ORI, oridonin; CD163, Cluster of Differentiation 163; CD206, Cluster of Differentiation 206; IFN-γ, Interferon-gamma; and TNF-α, Tumor necrosis factor-alpha.

**Figure 8:**
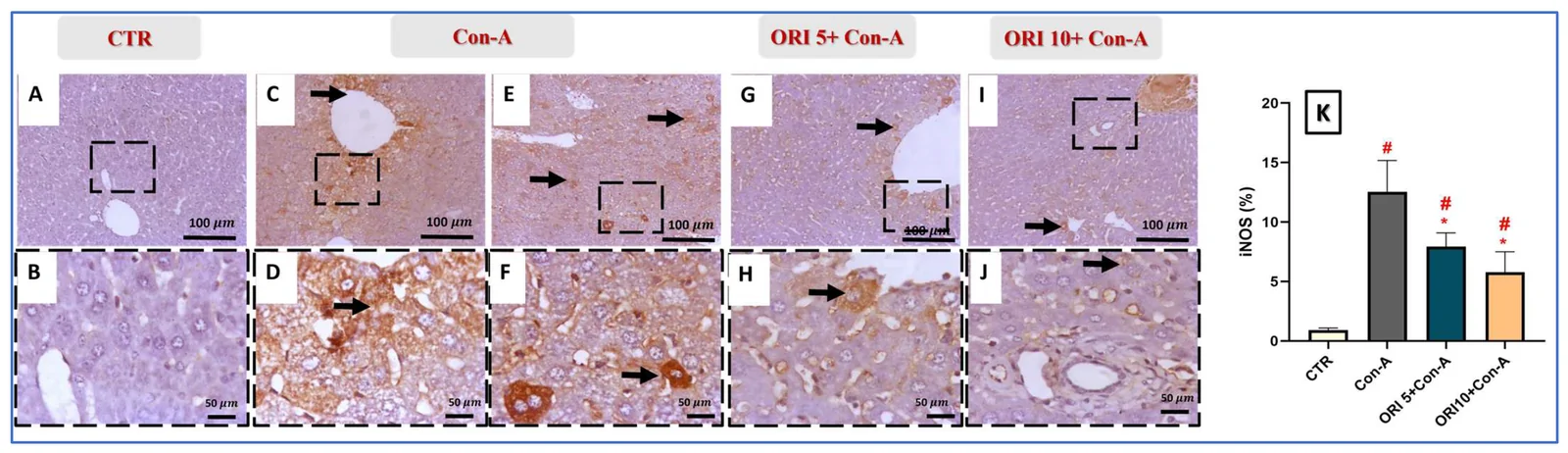
**Immunohistochemical analysis of the impact of ORI (5 and 10 mg/kg) on iNOS2 protein expression in the studied groups**. (**A**,**B**) CTR group showing negative immunostaining in hepatic parenchyma. (**C**–**F**) Con A showing focal to coalescing perivascular high immunopositive cytoplasmic expression in hepatocytes, as in (**C**,**D**), or diffuse scattered intense immunopositive cytoplasmic stained hepatocytes. (**G**,**H**) ORI 5 + Con A showing scattered mild cytoplasmic expression in hepatocytes around blood vessels and in hepatic parenchyma. (**I**,**J**) ORI 10 + Con A showing few immunopositive cytoplasmic-stained hepatocytes. Thin arrows = positive hepatocytes. Image magnification: 100× = Bar 100 μm, 400× = Bar 50 μm. (**K**) Semi-quantifications of % area protein expression of iNOS in the groups studied. Data were represented as mean ± SD using one-way ANOVA followed by Tukey–Kramer multiple comparisons, *n* = 5. # * Significant difference in relation to CTR and Con A, respectively. CTR, Control; Con A, Concanavalin A; ORI, Oridonin; and iNOS, inducible nitric oxide synthase.

To further elucidate the immunomodulatory effects of ORI in Con A-induced autoimmune hepatitis, hepatic CD163 and CD206 are assessed as associated pro-inflammatory cytokines and tissue-repair macrophage responses (Figure 7). Moreover, IFN-γ, TNF-α, and iNOS (Figure 8) were assessed among the experimental groups. CD163 and CD206, indicative of alternatively activated M2 macrophages which dampen inflammation and support tissue regeneration, were significantly decreased (*p* < 0.0001) after Con A treatment compared with that in the CTR group. Notably, ORI (5 and 10 mg/kg) pre-treatment preserved the normal CD163 levels (*p* = 0.0006 and *p* < 0.0001, respectively), and the high dose (10 mg/kg) succeeded in decreasing the Con A-triggered elevation in the CD206 protein level (*p* < 0.0001) and mRNA expression (*p* = 0.0128). These findings indicated that ORI drove M2 macrophage polarization, facilitating the resolution of hepatic inflammation and supporting tissue repair. Such an observed effect on M2 macrophage polarization was confirmed by the Con A-associated significant upsurge (*p* < 0.0001) in related pro-inflammatory cytokines including IFN-γ and TNF-α, and the elevated immunoexpression of iNOS in hepatic tissues as compared to those in CTR, indicating the activation of potent M2 macrophage polarization. Consequently, Con A induced an impaired cytokine milieu via T-cell-mediated liver inflammation and macrophage activation. Pre-treatment with ORI (5 and 10 mg/kg) prominently suppressed (*p* < 0.0001) IFN-γ, TNF-α, and iNOS expression, displaying potent anti-inflammatory and immunoregulatory effects in a dose-dependent manner.

### 3.7. Impact of ORI (5 and 10 mg/kg) on Hepatic Oxidant/Antioxidant Status

Considering the crucial role of oxidative stress in hepatocellular injury and immune-mediated inflammation, the potential of ORI to restore the redox balance and alleviate liver damage was investigated. As shown in Table 2, Con A treatment significantly increased oxidative stress markers, accompanied by a marked elevation in MDA content (*p* < 0.0001) and significant decrease in GSH and TAC levels (*p* < 0.0001), compared to the CTR group. ORI pre-treatment dose-dependently restored the TAC levels (5 mg/kg (*p* = 0.0011), 10 mg/kg (*p* < 0.0001)) and reinstated the GSH levels (*p* < 0.0001). Additionally, ORI significantly reduced lipid peroxidation, as indicated by the reduced MDA contents at both doses (*p* < 0.0001). These findings demonstrated that ORI effectively attenuated the oxidative stress in Con A-induced hepatic injury by enhancing antioxidant defenses and inhibiting lipid peroxidation.

### 3.8. Impact of ORI (5 and 10 mg/kg) on Hepatic TLR7 and PKM2

**Figure 9:**
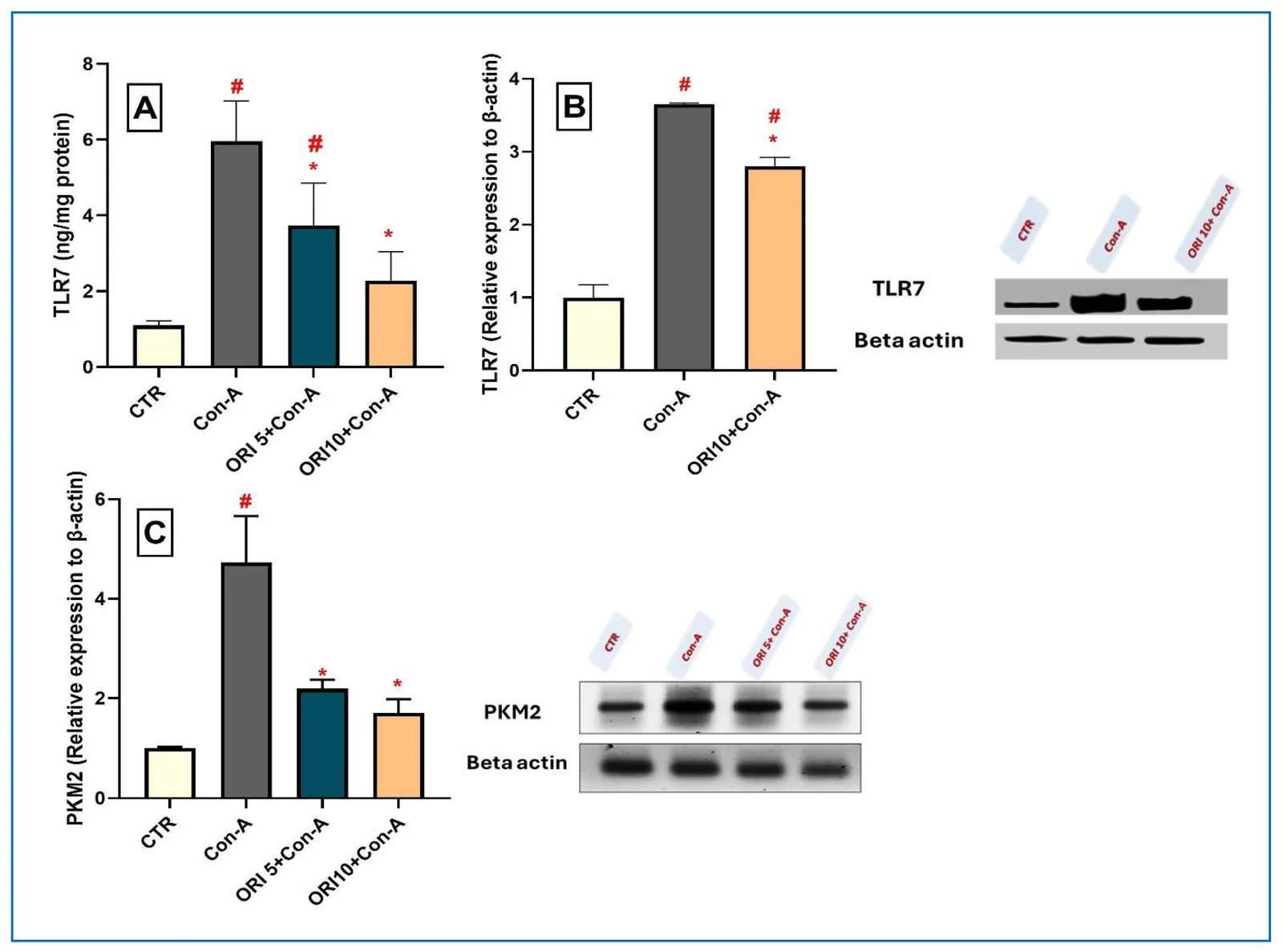
**Impact of ORI (5 and 10 mg/kg) on Con A-induced variations in the hepatic TLR7** (**A**) protein content**,** (**B**) relative protein expression to beta-actin, and representative Western blot bands**, and protein expression of PKM2** (**C**) relative protein expression to beta-actin and representative Western blot images. Data were represented as mean ± SD using one-way ANOVA followed by Tukey–Kramer multiple comparisons, *n* = 3–5. # * Significant difference in relation to CTR and Con, respectively. CTR, Control; Con A, Concanavalin A; ORI, oridonin; TLR7, toll-like receptor 7; and PKM2, M2 isoform of pyruvate kinase.

As shown in Figure 9, ORI significantly modulated key signaling pathways involved in immune-mediated injury and cellular stress in response to the Con A challenge. The protein levels of TLR7, an innate immunity receptor in liver tissues, and PKM2, an immune-metabolic regulator, were markedly elevated (*p* < 0.0001) in the Con A group compared to CTR. Pre-treatment with ORI (5 and 10 mg/kg) substantially reduced the expression of TLR7 (*p* = 0.0042 and *p* < 0.0001, respectively) and PKM2 (*p* = 0.0010 and *p* = 0.0003, respectively). Additionally, ORI at a high dose (10 mg/kg) attenuated the escalated protein expression of TLR7. These findings indicate its capacity to attenuate the Con A-induced activation of immune-mediated and stress-related signaling pathways.

### 3.9. Impact of ORI (5 and 10 mg/kg) on NLRP3 and IL-1β

**Figure 10:**
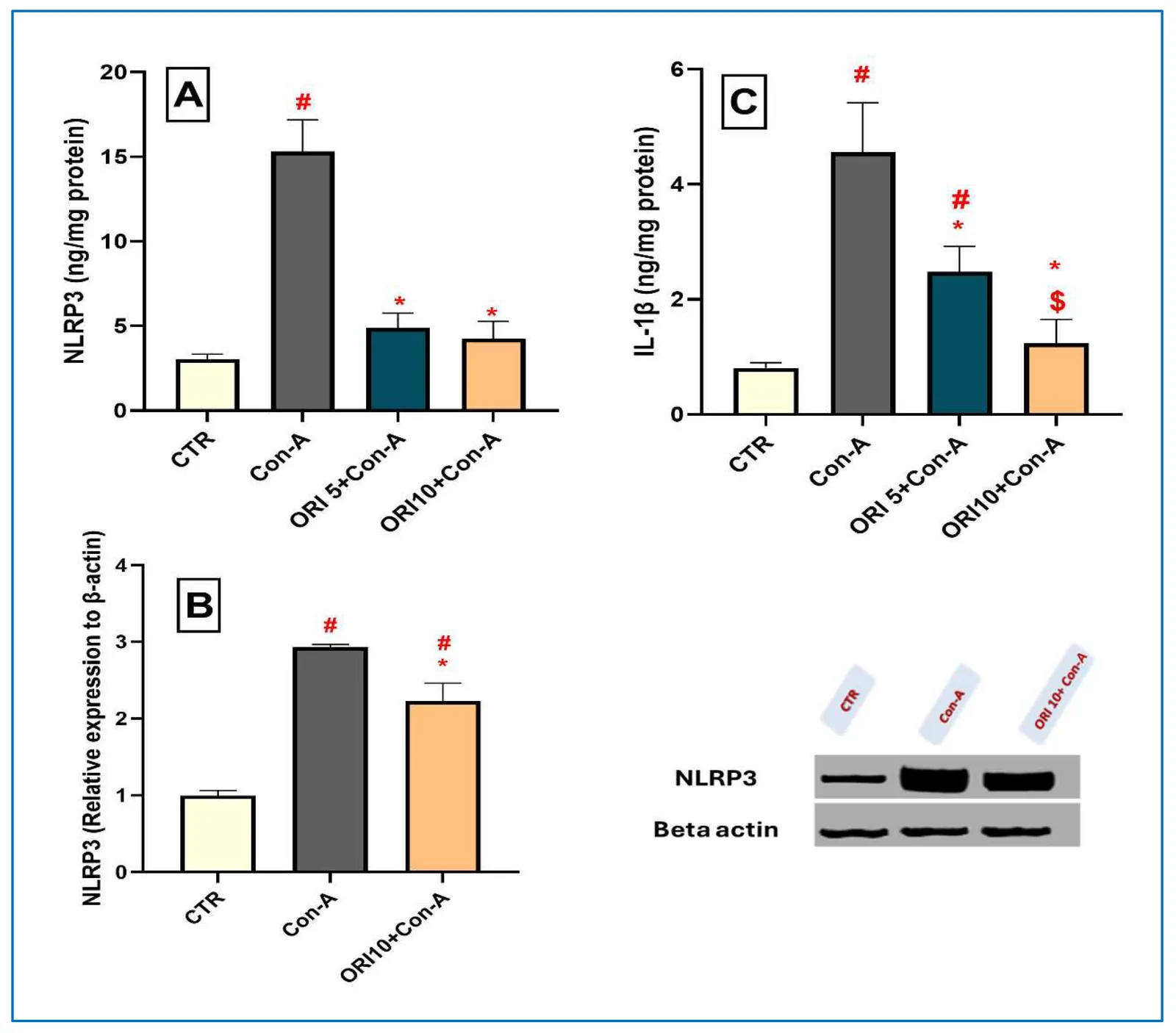
**Impact of ORI (5 and 10 mg/kg) on Con A-induced modifications in hepatic protein content of NLRP3** (**A**,**B**), relative protein expression to beta actin and representative Western blot bands, **and IL-1β** (**C**). Data were represented as mean ± SD using one-way ANOVA followed by Tukey–Kramer multiple comparisons, *n* = 3–5. # * $ Significant difference in relation to CTR, Con A, and ORI 5 + Con A, respectively. CTR, Control; Con A, Concanavalin A; ORI, oridonin; NLRP3, nucleotide-binding domain, leucine-rich-containing family, pyrin-domain-containing-3; and IL-1β, interleukin-1.

Following Con A administration, the hepatic protein levels of NLRP3 and IL-1β were significantly escalated (*p* < 0.0001), reflecting the activation of inflammatory pathways triggered by innate immune stimulation and subsequent metabolic reprogramming and oxidative stress (Figure 10). Pre-treatment with ORI (5 and 10 mg/kg) markedly suppressed these elevations in the levels of NLRP3 (*p* < 0.0001) and IL-1β (*p* = 0.0006 and *p* < 0.0001, respectively). Additionally, ORI at a high dose (10 mg/kg) attenuated the escalated protein expression of NLRP3. These findings demonstrate its potent anti-inflammatory effects.

## 4. Discussion

Given the rising incidence of AIH and limitations besides adverse effects associated with current immunosuppressive therapies, the identification of effective and safe natural compounds is of considerable clinical importance. Despite advances in the treatment of AIH [3,8]. AIH presents complex diagnostic and therapeutic challenges. In this context, the current study was designed to investigate the protective effects of ORI in a Con A-induced acute immune-mediated hepatitis model, which reproduces several immunological features of AIH.

Fortunately, our findings reveal that ORI exerts a notable hepatoprotective effect against Con A-induced liver injury, possibly linked to the M1/M2 macrophage polarization that coincided with the suppression of the TLR7/PKM2/NLRP3 inflammatory signaling pathway.

In line with previous observations [23,28], in the present study, Con A markedly increased serum AST and GGT activities, aligned with highly destructive histopathological changes with the semi-quantitative histopathological scoring analysis which corroborated the descriptive microscopic findings and provided objective evidence for the protective effects of ORI against Con A-induced hepatic injury, along with ultrastructural mitochondrial damage and the disruption of the rough endoplasmic reticulum with severe inflammatory infiltrates. These results together confirm the successful establishment of acute immune-mediated liver injury and illustrate the observed aggressive hepatotoxicity of T-cell and M1/M2 polarization-mediated inflammation.

The Con A-induced hepatitis initiated a large-scale immune response reflected by the activation of T-lymphocytes and macrophages. The activation of CD4^+^ T helper cells and CD8^+^ cytolytic T effector cells induces apoptotic cell death and necro-inflammation in hepatocytes through cytokine production. Macrophage activation plays a critical role in the progression of autoimmune liver injury [34]. Although macrophages are commonly described according to the M1/M2 polarization framework, the current evidence suggests that macrophage activation exists along a dynamic continuum of functional states rather than as two discrete phenotypes. Nevertheless, markers such as CD80 and CD86 are frequently associated with pro-inflammatory macrophage responses, whereas CD163 and CD206 are commonly linked to anti-inflammatory and tissue-repairing phenotypes [35,36]. In the present study, the hepatic CD4^+^ and CD8^+^ levels were significantly elevated by Con A, indicating the activation of surplus T-cell proliferation and infiltration. In accordance with these findings, Con A treatment dramatically increased the expression of M1 macrophage markers, including CD80 and CD86, while suppressing M2 macrophage markers, CD163 and CD206, resulting in a pronounced cytokine storm characterized by the elevation of pro-inflammatory cytokines, TNF-α, IFN-γ, and iNOS as well. These mediators primed inflammation, further enhancing T-cell activation and exacerbating hepatocellular necrosis. The cytokine profile in Con A-treated mice indicates a classical Th1-dominant immune response, characterized by elevated TNF-α and IFN-γ. These cytokines are critical in the pathogenesis of Con A-induced hepatitis by promoting hepatocyte apoptosis, neutrophil infiltration, and oxidative damage [37,38]. It should be noted that, although the Con A-induced hepatitis model is widely accepted for investigating immune-mediated liver injury and reproduces several immunological hallmarks of autoimmune hepatitis, including T-cell activation, cytokine overproduction, and hepatocellular damage, it primarily reflects an acute inflammatory response. Therefore, this model does not fully recapitulate the chronic and progressive nature of human AIH, which involves persistent autoimmunity, long-term immune dysregulation, and tissue remodeling. Consequently, the hepatoprotective effects of ORI observed in the present study should be interpreted within the context of acute experimental hepatitis, and further investigations using chronic AIH models are warranted to validate its therapeutic potential in human disease.

Notably, the observed triggered immune response and pro-inflammatory cytokines in Con A-injected mice promote oxidative stress, which consecutively amplifies hepatocellular injury and sustains the inflammatory cascade, resulting in hepatocyte necrosis [39,40]. In this perspective, in the current study, Con A increased hepatic MDA and depleted endogenous antioxidants, including GSH and TAC. These findings are consistent with previous reports in mice and cell lines [41,42].

In the current study, the role of the TLR7/PKM2/NLRP3 signaling axis was investigated for a better understanding and elucidation of molecular disturbances underlying Con A-induced liver injury, and, moreover, to clarify how innate immunity signaling links to adaptive T-cell-mediated hepatocellular injury and sustained hepatic inflammation.

TLRs serve as a key link between innate and adaptive immune responses [43], alongside their crucial regulatory role in the innate immune system, as they can detect not only invading pathogens, but also endogenous danger signals released by injured or necrotic cells [44]. They are expressed on various immune cells, including macrophages, monocytes, neutrophils, eosinophils, master cells, dendritic cells (DCs), and T cells [45].

The current study looks at the potential modulation of TLR7 in Con A-induced AIH, focusing on its role in amplifying inflammatory and oxidative stress, as TLR7 is involved in various immune-mediated diseases, among which is AIH [46]. The TLR 7/8 can activate the NF-κB pathway, leading to an elevation in inflammatory cytokines [47]. In accordance with [48]’s study, our findings proved that Con A administration significantly upregulated TLR7 expression, amplifying the enhanced hepatocellular damage.

There is a growing body of evidence that the activation of the NLRP3 inflammasome is a key contributor to immune-mediated liver injury in Con A-induced AIH. NLRP3 activation in hepatic macrophages upgrades caspase-1 cleavage and the maturation of IL-1β and IL-18. Afterwards, this cascade intensifies CD4^+^ T-cell responses, enhances macrophage M1 polarization, and exacerbates hepatic inflammation and hepatocyte death, and pyroptosis [6,49]. Moreover, TLR7 has been identified as an important upstream regulator of this process by providing the priming signal for NLRP3 activation through the NF-kB-dependent expression of NLRP3 and pro-IL-1β. Accordingly, the TLR7/NLRP3 axis is a critical inflammatory pathway linking innate immune sensing to inflammasome-mediated liver damage in AIH. In accordance with these findings, in the present study, TLR7/NLRP3/IL-1β expressions in the liver homogenate were significantly elevated, contributing to the observed hepatic injury and inflammation.

Notably, alongside the metabolic role of the M2 isoform of pyruvate kinase (PKM2), it acts as a key regulator of NLRP3 inflammasome assembly and activation, modulating pyroptosis in macrophages and representing a promising target for therapeutic intervention in acute liver failure (ALF) [7]. Concurrently, PKM2-driven metabolic reprogramming toward aerobic glycolysis, exacerbating mitochondrial dysfunction and oxidative stress, resulted from the upregulated TLR7 signaling. Consistently, our findings indicate that the observed increase in NLRP3 activation can be attributed, at least in part, to the PKM2 upregulation induced by the heightened TLR7 signaling, which subsequently led to an enhanced IL-1β release, further amplifying the hepatic inflammation and driving the macrophage polarization toward the pro-inflammatory M1 phenotype.

In the present study, ORI is the main pharmacological intervention against Con A-induced AIH. ORI was probed due to its well-established anti-inflammatory and immunomodulatory properties, its safety as a natural diterpenoid, and its recently recognized activity as a target of NLRP3, which represents the main core of our study [10,50,51].

The obtained results provide convincing evidence that ORI exerts notable protective effects against Con A-induced hepatic injury in mice. ORI markedly restored the Con A-triggered disturbances in liver enzymes, reduced the histopathological damage, and limited the hepatocyte damage. Likewise, the hepatic CD4^+^ and CD8^+^ levels were significantly decreased by ORI, indicating the associated suppression of Con A-triggered excessive T-cell proliferation and infiltration. This immunomodulatory effect is important as the dysregulated activation of T cells plays a pivotal role in the pathogenesis of AIH and is a key therapeutic target of established immunosuppressive therapies, that is, corticosteroids and azathioprine [8]. ORI also restored IgG synthesis by suppressing antigen presentation and reducing B-cell activation [11,52]. The M1/M2 polarization and related cytokine storm were dose-dependently ameliorated by ORI treatment, demonstrating the powerful inhibition of pro-inflammatory macrophage activation. Such a beneficial effect of ORI was reflected by the significant recovery of the CD163 and CD206 expression, the well-characterized anti-inflammatory M2 (repair-related) macrophage markers, and the suppression of CD80 and CD86 as M1-related macrophage markers. The effects described above as a whole suggest that ORI drives an M1 to M2 phenotypic shift, which is significantly related to the protective effect of ORI on liver injury [19,53]. As IFN-γ, TNF-α, and iNOS have all been shown to stimulate macrophage M1 differentiation as well, such cytokine inhibition observed here again confirms that ORI plays a role in modulating the dynamics of the macrophages. This observation reflects ORI’s capability to modulate the macrophage activity in inflammatory and immunological conditions. In accordance with our findings, the anti-inflammatory effect of ORI has been reported in diabetic neuropathy [54]. Overall, these results demonstrate that ORI may suppress cellular and humoral immune responses during AIH. ORI significantly decreased inflammatory cytokines, which suggested the attenuation of Th1 over-activation and immune-mediated hepatotoxicity [55]. Our findings are also consistent with the recent evidence demonstrating that ORI alleviates liver injury through the regulation of hepatic macrophages and the suppression of ROS/NLRP3 signaling. Together with the current results, these observations suggest that the modulation of macrophage-associated inflammatory pathways may represent a common mechanism underlying the hepatoprotective effects of ORI across different experimental settings [16]. Nevertheless, the present study extends the previous findings by examining ORI in an immune-mediated hepatitis model and evaluating its association with T-cell responses, macrophage polarization-associated markers, and TLR7/PKM2/NLRP3 signaling. Interestingly, the dose–response patterns differed among the investigated signaling molecules. While TLR7 and IL-1β exhibited a clearer dose-dependent reduction, PKM2 and NLRP3 showed comparable suppression at both ORI doses, suggesting that these targets may differ in their sensitivity to ORI or that maximal inhibition was achieved at the lower dose. These findings also raise the possibility that ORI exerts its hepatoprotective effects through multiple interconnected signaling pathways, which warrants further mechanistic investigation.

In line with previous studies [14,56], in addition to its observed immunomodulatory and anti-inflammatory actions, ORI exerted potential antioxidant effects, as evidenced by the reduced hepatic lipid peroxidation, and MDA content, and the restoration of endogenous antioxidant defenses, GSH and TAC. This antioxidant capacity of ORI is highly related to and associated with its suppression of inflammatory signaling, as excessive cytokine production and immune cell activation are major sources of ROS. Thus, ORI effectively interrupts the implied cycle between oxidative stress and immune-mediated hepatic inflammation, which is potentially involved in the overall hepatoprotective effect.

ORI suppressed the expression of key components within the inflammatory pathway, including TLR7, PKM2, and the subsequent inhibition of NLRP3 inflammasome assembly and activation, limiting IL-1β activation and, hence, release. These findings are in accordance with the results of previous studies of the hepatoprotective potential of ORI against ischemia–reperfusion injury by suppressing PKM2/NLRP3-mediated macrophage pyroptosis [15] and D-galactosamine (d-Gal)/lipopolysaccharide (LPS)-induced ALI [18] and carbon tetrachloride-induced liver fibrosis [57]. Interestingly, these effects resulted in the suppression of the cytokine storm and the modulation of the inflammatory milieu, shifting macrophage polarization away from the pro-inflammatory M1 phenotype toward the anti-inflammatory M2 phenotype, as discussed before, which collectively coincided with a reduced hepatic inflammation and enhanced hepatoprotection.

The present findings further support the growing interest in natural immunomodulatory compounds as potential therapeutic candidates for autoimmune liver diseases. Nevertheless, translating the experimental observations into clinical practice remains challenging. Although the Con A-induced hepatitis model reproduces several important immunological features of AIH, including T-cell activation, cytokine dysregulation, and immune-mediated hepatocellular injury, it does not fully reflect the complexity, chronicity, and heterogeneity of human disease. In addition, interspecies differences in immune responses, pharmacokinetics, and disease progression may influence the therapeutic outcomes. Therefore, while the current results suggest that ORI possesses promising hepatoprotective and immunoregulatory properties, further investigations are required in chronic and clinically relevant AIH models to confirm its long-term efficacy and safety. Future studies should also explore dose optimization, pharmacokinetic characteristics, and potential interactions with currently used immunosuppressive therapies before a clinical evaluation in patients with AIH can be considered.

## 5. Conclusions

In conclusion, ORI protected against Con A-induced autoimmune hepatitis and attenuated liver injury through the modulation of the inflammatory, oxidative, and immune responses. ORI reduced T-cell activation, altered the macrophage-polarization-associated markers, attenuated cytokine production, and improved the hepatic redox balance. These protective effects were accompanied by a decreased expression of TLR7, PKM2, and NLRP3 signaling components, suggesting a potential involvement of this inflammatory pathway in the observed hepatoprotection. Collectively, the present findings support the hepatoprotective and immunomodulatory potential of ORI in an experimental model of autoimmune hepatitis. However, given the acute nature of the Con A model and the absence of dedicated safety, pharmacokinetic, and long-term efficacy assessments, further mechanistic studies, chronic disease models, and translational investigations are required before clinical relevance can be established.

### Limitations and Future Prospects

Despite the promising findings of the present study, several limitations should be acknowledged. First, the current investigation was conducted using an acute Con A-induced mouse model of AIH, which, although widely utilized, may not fully represent the complexity and chronic progression of human AIH. Additionally, this study evaluated the protective and immunomodulatory potential of the short-term administration of ORI, not the long-term therapeutic outcomes or potential safety aspects of prolonged administration. Future studies are needed to assess the post-treatment effects of ORI and to validate these findings on long-term administration against a chronic AIH model. Ultimately, translational and preclinical investigations are required to determine the pharmacokinetic profile, safety, and clinical applicability of ORI as a potential therapeutic candidate for human AIH. Second, the precise molecular interactions within the current study were explored using ELISA, IHC, Western blot, and quantitative RT-PCR; however, future studies including flow cytometry and specific inhibitors or gene-silencing approaches, such as siRNA or CRISPR, would provide more definitive mechanistic confirmation. Exploring gender-related differences remains important for translational relevance, and investigating the effects of ORI in female mice represents a valuable direction for future studies. Another limitation relates to the translational applicability of ORI itself. Although ORI demonstrated promising hepatoprotective and immunomodulatory effects in the present study, its clinical development remains challenging because of its poor aqueous solubility, limited oral bioavailability, and suboptimal pharmacokinetic characteristics. Previous studies have highlighted the need for optimized formulations, novel delivery systems, or structural modifications to enhance its therapeutic utility and systemic exposure. Therefore, future investigations should focus not only on validating the biological efficacy of ORI in chronic disease models but also on improving its pharmaceutical properties to facilitate potential clinical translation. An added limitation of the present study is that only two doses of oridonin (5 and 10 mg/kg) were evaluated. Although both doses exerted significant immunomodulatory effects, a clear dose-dependent response was not consistently observed for all measured parameters, particularly CD80 and CD86 expression. Therefore, the minimum effective dose and the complete dose–response relationship could not be established. Future studies incorporating lower doses of oridonin are warranted to better define its pharmacological profile and optimize its therapeutic dosing.

## Figures and Tables

**Table 1:** Impact of ORI (5 and 10 mg/kg) on Con A-induced changes in serum liver enzyme activities.

Group	AST (U/L)	GGT (U/L)
**CTR**	139.83 ± 19.34	10.33 ± 2.07
**ORI-CTRL**	140.83 ± 9.39	10.50 ± 1.05
**Con A**	593.50 ± 75.20 #	91 ± 4.56 #
**ORI 5 + Con A**	410.67 ± 17.22 # *	66.83 ± 4.54 # *
**ORI 10+ Con A**	252.50 ± 33.50 # * $	40 ± 1.90 # * $

Values were presented as mean ± SD. Data were obtained by one-way ANOVA followed by Tukey–Kramer multiple comparisons, *n* = 6. # * $ Significant compared to CTR, Con A, and ORI 5+ Con A, respectively. AST, aspartate aminotransferase; GGT, gamma-glutamyl transferase; CTR, Control; Con A, Concanavalin A; and ORI, Oridonin.

**Table 2:** Impact of ORI (5 and 10 mg/kg) on Con A-induced variations in hepatic oxidative milieu.

Group	MDA (nmol/mg Protein)	GSH (mmol/mg Protein)	TAC (nmol/mg Protein)
**CTR**	72.42 ± 4.85	336.02 ± 13.05	5.505 ± 0.74
**ORI-CTR**	77.33 ± 2.53	331.6 ± 16.85	5.375 ± 0.66
**Con A**	167.22 ± 13 #	114.14 ± 2.10 #	0.8725 ± 0.09 #
**ORI 5 + Con A**	101.74 ± 6.12 # *	307.15 ± 6.62 # *	2.798 ± 0.20 # *
**ORI 10+ Con A**	92.24 ± 6.67 # *	318.84 ± 10.61 # *	4.348 ± 0.69 # * $

Data were represented as mean ± SD using one-way ANOVA followed by Tukey–Kramer multiple comparisons, *n* = 6. # * $ Significant difference in relation to CTR, Con A, and ORI 5 + Con A, respectively. CTR, Control; Con A, Concanavalin A; ORI, oridonin; GSH, reduced glutathione; MDA, malondialdehyde; and TAC, total antioxidant.

## Data Availability

The original contributions presented in this study are included in the article/Supplementary Materials. Further inquiries can be directed to the corresponding author.

## Supplementary Materials

The following supporting information can be downloaded at: https://www.mdpi.com/article/10.3390/jox16040138/s1, File S1: Supplementary information for microscopic images corresponding to Figure 1, Figure 2, Figure 5, Figure 6, and Figure 8; File S2: Supplementary information for Western blot images corresponding to Figure 7, Figure 9, and Figure 10; Supplementary Table S1: Biomarkers and their corresponding assay kits; Supplementary Table S2: Sequences of forward and reverse primers used for real-time quantitative PCR analyses.

## Abbreviations

AIH	Autoimmune hepatitis
AST	Aspartate aminotransferase
CD	Cluster of differentiation
Con A	Concanavalin A
GGT	Gamma-glutamyl transferase
GSH	Reduced glutathione
IFN-γ	Interferon gamma
IgG	Immunoglobulin G
IL-1β	Interleukin-1 beta
iNOS	Inducible nitric oxide synthase
MDA	Malondialdehyde
NF-κB	Nuclear factor kappa B
NLRP3	NOD-like receptor family pyrin domain containing 3
ORI	Oridonin
PKM2	Pyruvate kinase M2
ROS	Reactive oxygen species
TAC	Total antioxidant capacity
TLR7	Toll-like receptor 7
TNF-α	Tumor necrosis factor alpha
