# Supplementary material for: Oridonin Ameliorates Concanavalin A-Elicited Hepatitis in Mice: Insight into Suppressing TLR7/PKM2/NLRP3-Driven Inflammation and M1/M2 Polarization
Source: J Xenobiot. 2026 Jul 28;16(4):138. doi: 10.3390/jox16040138 (PMC13514674; doi:10.3390/jox16040138)
Supplement: Supplementary file 1 [file jox-16-00138-s001.zip › FileS2-Supplementary information for western blot images corresponding to Figures 7,9,and 10/Figure 10/NLRP3.pdf]

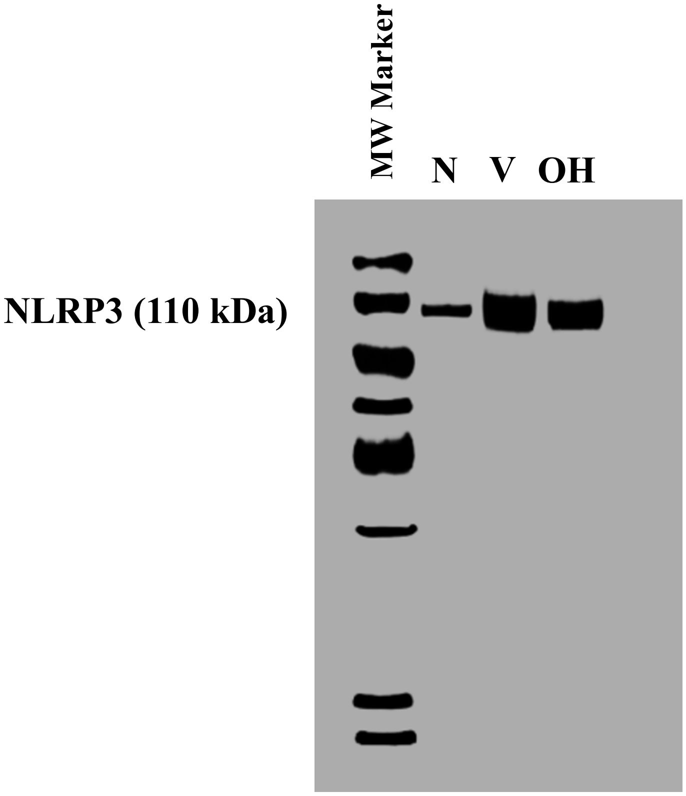

NLRP3

N : Control V: Con-A OH: ORI 10+ Con-A

Beta actin 43 kDa

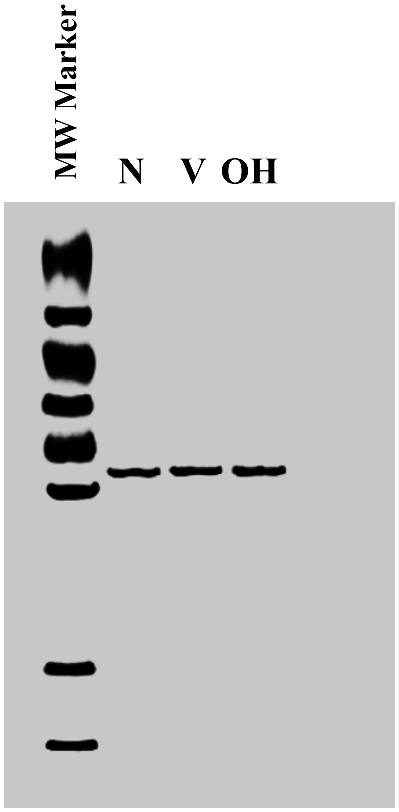

Beta actin

N : Control **V: Con-A** **OH: ORI 10+ Con-A**
