# Supplementary material for: Oridonin Ameliorates Concanavalin A-Elicited Hepatitis in Mice: Insight into Suppressing TLR7/PKM2/NLRP3-Driven Inflammation and M1/M2 Polarization
Source: J Xenobiot. 2026 Jul 28;16(4):138. doi: 10.3390/jox16040138 (PMC13514674; doi:10.3390/jox16040138)
Supplement: Supplementary file 1 [file jox-16-00138-s001.zip › FileS2-Supplementary information for western blot images corresponding to Figures 7,9,and 10/Figure 7/~WRL0892.tmp]

**OH**

**N1**

**V**

**CD206 (---kDa)**

**CD206 Run 1**

**N : Control	V: Con-A	OH: ORI 10+ Con-A**

**OH**

**N1**

**V**

**CD206 (---kDa)**

**CD206 Run 2**

**N : Control	V: Con-A	OH: ORI 10+ Con-A**

**OH**

**N1**

**V**

**CD206 (---kDa)**

**CD206 Run 3**

**N : Control	V: Con-A	OH: ORI 10+ Con-A**

**OH**

**V**

**N**

**Beta actin 43 kDa**

**Beta actin**

**N : Control	V: Con-A	OH: ORI 10+ Con-A**
