# Supplementary material for: Oridonin Ameliorates Concanavalin A-Elicited Hepatitis in Mice: Insight into Suppressing TLR7/PKM2/NLRP3-Driven Inflammation and M1/M2 Polarization
Source: J Xenobiot. 2026 Jul 28;16(4):138. doi: 10.3390/jox16040138 (PMC13514674; doi:10.3390/jox16040138)
Supplement: Supplementary file 1 [file jox-16-00138-s001.zip › FileS2-Supplementary information for western blot images corresponding to Figures 7,9,and 10/Figure 9/TLR7.pdf]

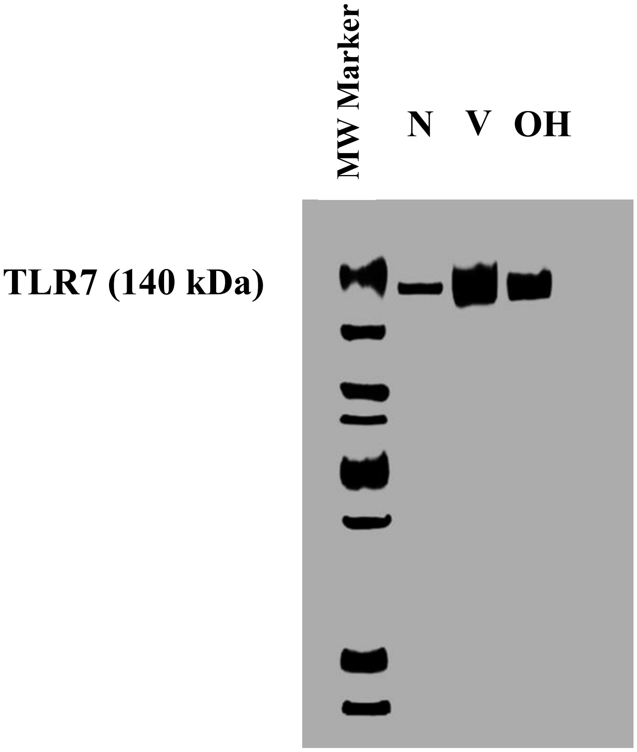


**TLR7**

**N : Control** **V: Con-A** **OH: ORI 10+ Con-A**

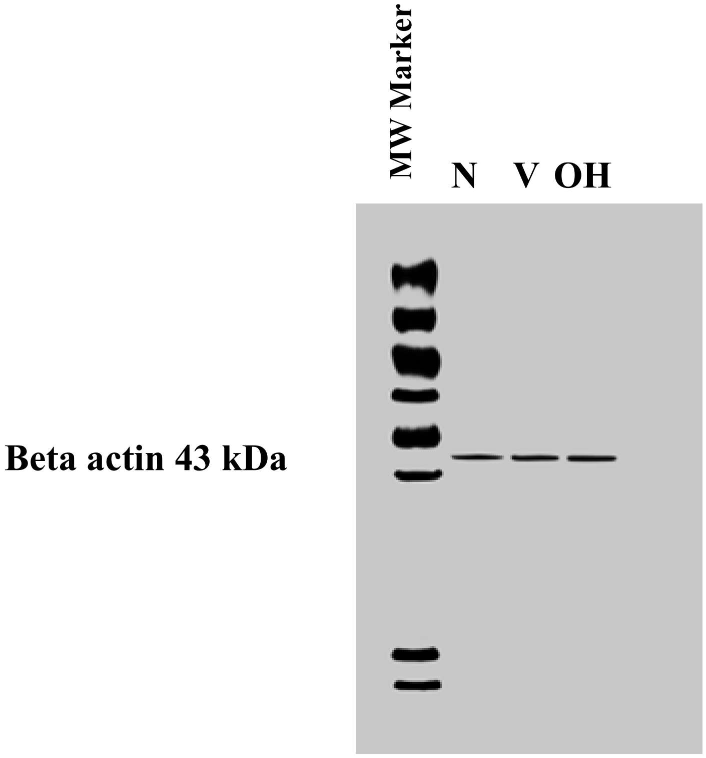


**Beta actin**

**N : Control V: Con-A OH: ORI 10+ Con-A**
