# Supplementary material for: Oridonin Ameliorates Concanavalin A-Elicited Hepatitis in Mice: Insight into Suppressing TLR7/PKM2/NLRP3-Driven Inflammation and M1/M2 Polarization
Source: J Xenobiot. 2026 Jul 28;16(4):138. doi: 10.3390/jox16040138 (PMC13514674; doi:10.3390/jox16040138)
Supplement: Supplementary file 1 [file jox-16-00138-s001.zip › Table S1 and S2.pdf]

Table S1. Biomarkers and their corresponding assay kits.

Biomarker	Assay kit
Cluster of differentiation (CD)- 4	MyBiosource, USA, Cat# MBS2506108
Cluster of differentiation (CD)- 8	MyBiosource, USA, Cat# MBS2516027
Immunoglobulin G (IgG)	Elabscience, USA, Cat# E-EL-M0692
Tumor necrosis factor (TNF)-α	MyBiosource, USA, Cat# MBS825075
Cluster of differentiation (CD)-163	MyBiosource, USA, Cat# MBS2516344
Interferon (IFN)-γ	Elabscience, USA, Cat# E-EL-M0048
Cluster of differentiation (CD)-80	MyBiosource, USA, Cat# MBS825050
Cluster of differentiation (CD)-86	LSBio, USA, Cat# LS-F15288
Nucleotide-binding domain, leucine-rich–containing family, pyrin domain–containing-3 (NLRP3)	MyBiosource, USA, Cat# MBS7606270
Toll-Like Receptor 7 (TLR7)	ELK Biotechnology, China, Cat# ELK2700
IL-1β (Interleukin 1 Beta)	FineTest®, , China, Cat# EM0109

Table S2. Sequences of forward and reverse primers used for real time quantitative PCR analyses.

Primer		Sequence
**CD86**	Forward	ACGATGGACCCCAGATGCACCA
	Reverse	GCGTCTCCACGGAAACAGCA
**CD206**	Forward	TCAGCTATTGGACGCGAGGCA
	Reverse	TCCGGGTTGCAAGTTGCCGT
**GAPDH (Reference Gene)**	Forward	TGTGTCCGTCGTGGATCTGA
	Reverse	TTGCTGTTGAAGTCGCAGGAG

CD86; Cluster of differentiation 86, CD206; Cluster of differentiation 206, GAPDH; glyceraldehyde-3-phosphate dehydrogenase.
